# The importance of estimating the burden of disease from foodborne transmission of *Trypanosoma cruzi*

**DOI:** 10.1371/journal.pntd.0011898

**Published:** 2024-02-08

**Authors:** Lucy J. Robertson, Arie H. Havelaar, Karen H. Keddy, Brecht Devleesschauwer, Banchob Sripa, Paul R. Torgerson

**Affiliations:** 1 Parasitology, Department of Paraclinical Sciences, Faculty of Veterinary Medicine, Norwegian University of Life Sciences, Ås, Norway; 2 Emerging Pathogens Institute, Global Food Systems Institute, Animal Sciences Department, University of Florida, Gainesville, Florida, United States of America; 3 Independent consultant, Johannesburg, South Africa; 4 Department of Epidemiology and Public Health, Sciensano, Brussels, Belgium; Department of Translational Physiology, Infectiology and Public Health, Ghent University, Merelbeke, Belgium; 5 Tropical Disease Research Center, Department of Tropical Medicine, Faculty of Medicine, Khon Kaen University, Khon Kaen, Thailand; 6 Section of Epidemiology, Vetsuisse Faculty, University of Zürich, Zürich, Switzerland; Centre for Tropical Diseases, ITALY

## Abstract

Chagas disease (ChD), caused by infection with the flagellated protozoan, *Trypanosoma cruzi*, has a complicated transmission cycle with many infection routes. These include vector-borne (via the triatomine (reduviid bug) vector defecating into a skin abrasion, usually following a blood meal), transplacental transmission, blood transfusion, organ transplant, laboratory accident, and foodborne transmission. Foodborne transmission may occur due to ingestion of meat or blood from infected animals or from ingestion of other foods (often fruit juice) contaminated by infected vectors or secretions from reservoir hosts. Despite the high disease burden associated with ChD, it was omitted from the original World Health Organization estimates of foodborne disease burden that were published in 2015. As these estimates are currently being updated, this review presents arguments for including ChD in new estimates of the global burden of foodborne disease. Preliminary calculations suggest a burden of at least 137,000 Disability Adjusted Life Years, but this does not take into account the greater symptom severity associated with foodborne transmission. Thus, we also provide information regarding the greater health burden in endemic areas associated with foodborne infection compared with vector-borne infection, with higher mortality and more severe symptoms. We therefore suggest that it is insufficient to use source attribution alone to determine the foodborne proportion of current burden estimates, as this may underestimate the higher disability and mortality associated with the foodborne infection route.

## Author summary

The World Health Organization is working to update previous estimates of the global health burden associated with different foodborne diseases. Previous estimates, published in 2015, did not include Chagas Disease (ChD). This disease, which occurs predominantly in Latin America, is caused by a parasite, *Trypanosoma cruzi*. The disease can be transmitted to people by a variety of routes. Although the conventionally accepted pathway is via an insect vector, foodborne transmission seems to be very important. This can be via contaminated food, particularly fruit juice or by eating undercooked meat from infected animals.

Here, we argue why this parasitic infection should be included in the new estimates of the health burden of foodborne diseases. Growing evidence indicates that foodborne transmission of *T*. *cruzi* occurs as commonly as vector-borne infection. Moreover, foodborne infection results in considerably more severe disease than vector-borne infection, with higher mortality and greater symptom severity, particular in the acute phase.

## 1. Background

*Trypanosoma cruzi*, a flagellated protozoan parasite (class Kinetoplastida), is the etiological agent of Chagas disease (ChD), also known as American trypanosomiasis (trypanosomosis), a serious, potentially fatal, infection. An estimated 6 to 7 million people are infected with *T*. *cruzi* worldwide, with about 10,000 deaths annually (https://www.who.int/news/item/14-04-2022-world-chagas-disease-day-bringing-a-forgotten-disease-to-the-fore-of-global-attention; website updated in April 2022). Although this infection occurs predominantly in Latin American countries, the incidence in other global regions, particularly North America and Europe, is rising; cases outside Latin America are largely associated with migrants from endemic countries bringing the infection with them [1].

Transmission of this disease is complex and involves multiple pathways elucidated in greater detail below. Whereas the conventionally accepted (“traditional”) route of transmission is vector-borne, oral transmission, potentially by contaminated food or drink, seems to be of increasing importance and becoming more recognized. This is partly because vector-borne transmission is being reduced by initiatives such as improved housing; simultaneously, there is greater human encroachment on wilderness areas, resulting in closer contact with sylvatic transmission routes [2].

In 2006, the World Health Organization (WHO) developed a strategy to estimate the global burden of foodborne diseases according to etiological agent and constituted an expert group (The Foodborne Disease Burden Epidemiology Reference Group (FERG)) to advise on this task. The first estimates were published in 2015 [3]. In 2021, WHO decided that there was a requirement to update the estimates based on more recent data and with an extended list of etiological agents. In order to undertake this task, FERG membership was reconstituted (FERG for 2021 to 2025), with some of the previous team and some new members, to oversee collection of relevant data for this global effort.

In the 2015 FERG estimates, *T*. *cruzi* was excluded from the list of parasites due to lack of resources [4]. A subsequent publication noted that, as the focus of the foodborne burden estimates was on global impact, some important pathogens with restricted distribution were not included [5].

Indeed, a crude-level calculation of the disability-adjusted life-years (DALYs) associated with foodborne ChD—based on ChD burden reported in Global Burden of Diseases (GBD) [6] and data on the proportion of cases that are likely orally transmitted—gave an estimate of 273,000 DALYs per annum attributable to foodborne transmission globally [5]. This exceeds the DALYs associated with foodborne transmission of several of the other pathogens that were included in the first FERG estimates (see Section 4; [3,4,7]). Given that the vast majority of the ChD burden is restricted to Latin America, the burden per 100,000 of exposed population living in the endemic region is even higher. Further crude estimates based on more recent GBD data [1] are discussed in Sections 4 and 6.

The question now arises on whether and how *T*. *cruzi* should be included in this new round of estimating the global burden of foodborne disease. It is clear that although foodborne ChD is gaining recognition, the importance of the foodborne infection route is not widely understood. Indeed, many articles continue to be published in which oral/foodborne transmission is not included in the introductory text that describes the parasite, the disease, and infection routes (e.g., [8–10]), or describes oral transmission as being an infrequent infection route (e.g., [11]). Even the current English Wikipedia page states that vector-borne transmission is “the parasite’s only transmission route”; also today’s Spanish and Portuguese Wikipedia pages only describe vector-borne transmission.

More importantly, the WHO Road Map for neglected tropical diseases 2021 to 2030 [12] identifies only 4 transmission routes (vectoral, transfusion, transplantation, and congenital) in the first table regarding disease-specific targets. WHO lists 5 main objectives for eliminating ChD by 2030. These are: verification of interruption of vectorial domiciliary transmission; verification of interruption of transmission by blood transfusion; verification of interruption of transmission by organ transplantation; elimination of the congenital form of the disease; and 75% coverage of antiparasitic treatment of the eligible population. None of these addresses the importance of foodborne transmission.

## 2. Methods

Here, we describe the transmission routes of *T*. *cruzi* to people and, by a narrative review of the literature, discuss why foodborne ChD should be considered separately in terms of disease burden. We also explain why estimates of the ChD burden will be higher than previously calculated when clinical differences associated with oral transmission are taken into account. One limitation of our purposive approach is that, by necessity, not all relevant studies and results are mentioned.

## 3. Routes of transmission of ChD

**Fig 1:**
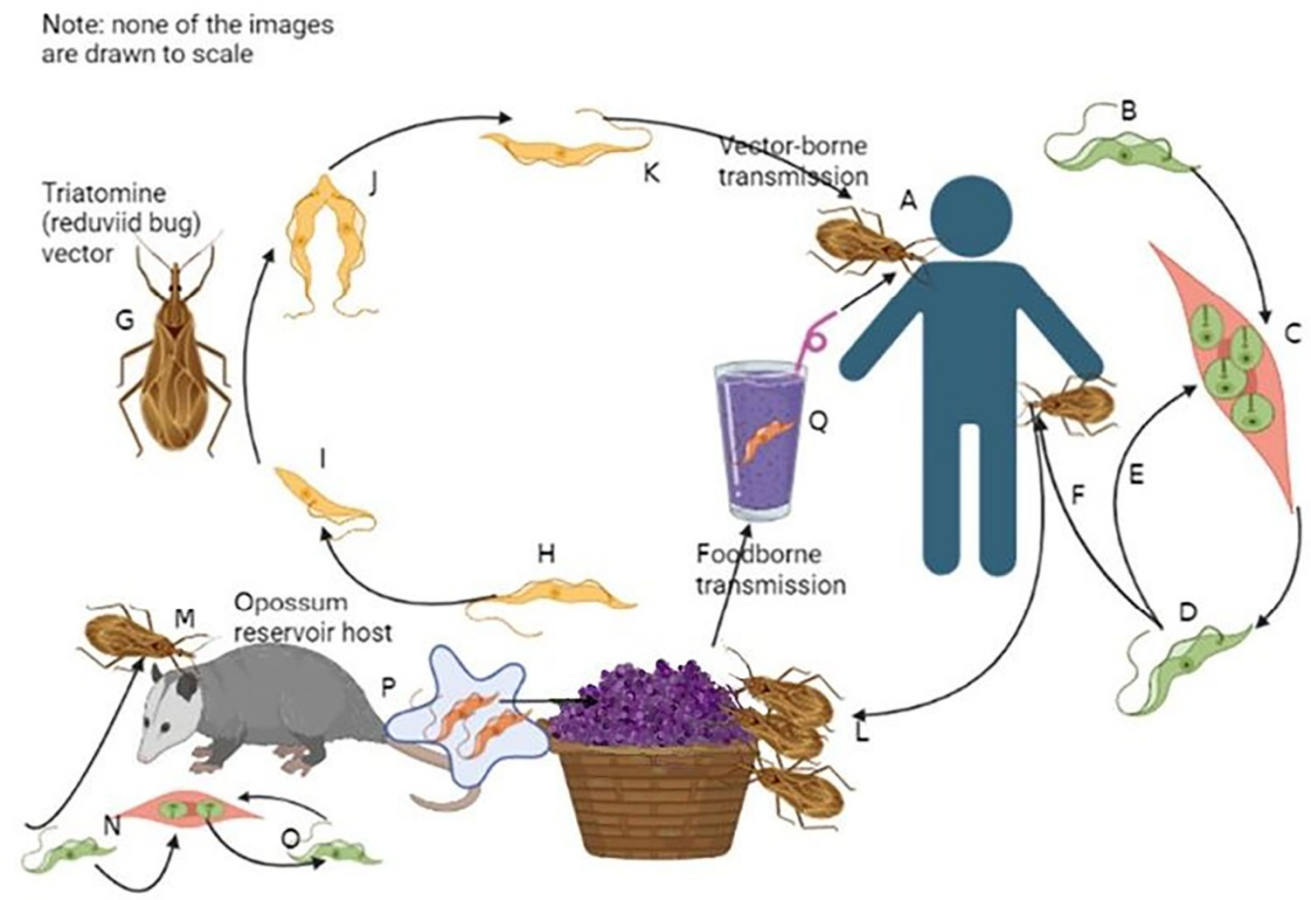
Lifecycle of *T*. *cruzi* demonstrating the predominant infection routes of people via vector-borne and foodborne transmission. In this illustration, food is contaminated either via an infected triatomine (reduviid bug) or via secretions from an infected reservoir host. Note: Fig 1 was initially created in BioRender (www.biorender.com) and then modified. Less frequent transmission routes (not included in this figure) are: transplacental/congenital infection, blood transfusion and organ/bone marrow transplant infection, and laboratory accidents. In addition, consumption of inadequately cooked meat or blood from infected animals may act as a transmission route. An infected triatomine vector takes a blood meal from a person and infects them by defecating in or near to the wound with trypomastigotes in its feces (**A**). The trypomastigotes (**B**) invade cells and differentiate into intracellular amastigotes (**C**) that multiply by binary fission. These differentiate into trypomastigotes that are released into the circulation (**D**) and infect further cells (**E**) from various tissues, where they again transform into intracellular amastigotes and replicate (**C**). The triatomine vector becomes infected by feeding on an infected person (**F**). Inside the midgut of the vector (**G**), the ingested trypomastigotes (**H**) transform into epimastigotes (**I**). Here they multiply, before differentiating into infective metacyclic trypomastigotes in the hindgut (**K**) that may then be defecated into a feeding wound (**A**). Food, particularly fruit, may also be contaminated by being colonized by infected triatomines (**L**). In addition, reservoir hosts (such as opossums) can also be infected from trypomastigotes in the feces of feeding vectors (**M**). The circulating trypomastigote-intracellular amastigote cycle (**N, O**) then occurs. Food may be contaminated by trypomastigotes in the secretions of the opossums (**P**). Food, particularly fruit juices, contaminated by trypomastigotes (**Q**), either via infected triatomines (**L**) or secretions from infected opossums (**P**), may act as a vehicle for oral infection.

The lifecycle of *T*. *cruzi* is relatively complicated and includes various routes of infection for ChD (see Fig 1). These are:

1. Vector-borne: This transmission route is often considered the “traditional” infection path in endemic areas and is related to the ability of different triatomine (reduviid bug) species to adapt to, and colonize, different ecotopes, particularly home environments [13]. During transmission, an infected vector defecates into a wound (usually, but not always, a bite wound from the vector itself), introducing infective trypomastigotes. These invade host cells, where they replicate to non-flagellated amastigote forms that may remain quiescent and intracellular, or may develop to trypomastigotes that return to the bloodstream. From here they can both infect other cells and be acquired by the vector. In addition, vectors may contaminate food; this is considered under foodborne transmission in this list (point 6b).
2. Transplacental/congenital: This usually occurs after the 12th week of gestation, when the maternal blood supply becomes diffuse and continuous in the entire placenta; high maternal parasitemia is a proposed risk factor, and transmission is thus more likely to occur when infection is acquired during pregnancy (acute phase of disease). Coinfection with HIV and/or *Plasmodium vivax* is associated with increased risk of congenital transmission [14].
3. Blood transfusion: Apart from plasma derivatives, all blood components from an infected donor may transmit *T*. *cruzi* and the parasite remains viable for considerable periods at room temperature, refrigerated, and frozen. Although only a few hundred cases have been published, in some places (particularly where the parasite is not endemic), it can be an important transmission route. Transmission likelihood from an infected donor depends on various factors, including: extent of parasitemia at donation, transfusion volume, strain, recipient immune status. This transmission route can be mitigated against by use of low-risk donors (questionnaires, donor screening) and use of pathogen-reduction technologies on donated blood components, e.g., UV irradiation [15–16].
4. Organ/bone marrow transplant: This transmission route is less common than blood transfusion transmission, as organ transplants occur less frequently and screening mitigates against this infection route. However, a lack of suitable donated organs has encouraged use of organs from infected donors. Reactivation of *T*. *cruzi* in donated organs is likely exacerbated by immunosuppression to limit rejection of the transplant and seems to vary according to organ and degree of immunosuppression [11].
5. Laboratory accident: The predominant stage of *T*. *cruzi* in laboratory culture is the non-infectious epimastigote stage, although infective trypomastigotes occur, as well as intermediate forms (transitional epimastigotes) that are also infectious [17]. Despite occasional reports of laboratory-acquired *T*. *cruzi* infections, predominantly from a needle or other sharp object that pierced the skin [18] (but also from handling of infected mammals; [19]), this is the least common transmission route. However, it can have severe consequences, and at least one of 65 laboratory-accident cases has proven fatal [18].
6. Foodborne/oral: Although it has been argued that foodborne/oral transmission is another form of vector-borne transmission [20], this transmission route does not necessarily involve triatomines and is therefore more usefully considered separately. In addition, even when triatomines are involved in foodborne transmission, the entry site differs (skin abrasion versus digestive tract), and this can be of pathophysiological and clinical relevance.

The 3 main routes of foodborne transmission are [21]:

- Meatborne transmission: Ingestion of inadequately cooked meat or organs from animals infected with *T*. *cruzi* has been indicated as a transmission route, but in many cases, it is very difficult to exclude other infection routes. A systematic review of ChD transmission from consumption of game meat [22] identified only 5 reports (in 6 papers) where transmission due to ingestion of meat seemed likely. Results from 2 different animal experiments [23,24], although superficially contradictory, indicate that inadequately cooked bloody meat containing trypomastigotes could be a source of infection, but non-bloody muscle, tissue, and organs seem less likely to be so [21]. ChD infection transmitted by drinking the blood of wild animals (e.g., armadillos) as a part of religious rituals or traditional medicine has been reported from rural areas of Latin America [25].
- Contamination of food by infected vectors: This seems to be the most common oral infection route and has thus resulted in oral transmission being considered an extended part of vector-borne transmission. This is unfortunate as the “traditional” vector-borne route results in a different clinical picture, and therefore, the 2 routes cannot be considered as one and the same. For oral transmission, fruit juices tend to be the most common, but not the only, vehicle (other products reported as vehicles include sugarcane juice, ice cream, soup, and shrimps). Food contamination is either with the feces of the vectors or, more commonly, particularly for juices, the entire vector is incorporated into the food product. As triatomine nymphs are only a couple of millimetres in size, they may be overlooked in preparation of juices [21,25].
- Contamination of food by infected marsupials: Different species of opossum, particularly *Didelphis marsupialis* (common opossum), are common reservoirs of *T*. *cruzi*. When these marsupials are infected with *T*. *cruzi*, in addition to trypomastigotes circulating in blood and amastigotes colonizing tissues, the parasite will reproduce and transition to metacyclic trypomastigote within the opossum’s anal gland secretions. The lifecycle is thereby completed in the absence of the triatomines. The parasites are infective when expelled in aerosolized secretions, and contamination of food may occur when *Didelphis* spp. have access to open kitchens with exposed foods. This transmission pathway has been suggested (but not proven) to have occurred during outbreaks in which the oral route is suspected, but triatomines were apparently absent and infected opossums were present [26–28].

## 4. More severe clinical outcomes suggest that foodborne ChD should be considered separately from ChD transmitted by other routes of infection in estimating the burden of disease

Burden of disease is usually presented as DALYs, which includes both reduction in life expectancy and diminished years of healthy life [29]. Thus, the DALY burden for a particular condition is the sum of YLL (years of life lost due to premature mortality) and YLD (years lived with disability, weighted for the severity of the illness) as spread across a population (often global or national).

Many pathogens that were included in the 2015 FERG estimates could be transmitted both by food and other transmission routes. In order to estimate the burden associated with the component associated with foodborne transmission, first the entire DALY burden due to all cases of the specific hazard was estimated. The proportion of cases associated with foodborne transmission was then estimated [30], and incidence, mortality, and DALYs associated with foodborne transmission could then be calculated, assuming that clinical outcomes were independent of transmission route. Estimating DALYs is difficult and with considerable uncertainty for all pathogens, and estimating the proportion that is foodborne (for those pathogens that are not solely foodborne) is even more challenging. For various pathogens that were covered in the FERG estimates for 2015, including parasites such as *Echinococcus* and intestinal protozoa, a structured expert elicitation approach was used to estimate the proportion of cases likely to be foodborne [30]. The data indicate substantial uncertainty (uncertainty from individual experts and disagreement between experts) for most parasites and very high uncertainty for others (e.g., *Echinococcus multilocularis*).

Thus, for example, for ascariosis (disease associated with *Ascaris* infection), the total global burden of DALYs was estimated to be 1,317,535 (95% uncertainty interval (UI): 1,182,187 to 2,700,572), whereas the total global burden of DALYs due to foodborne transmission was estimated to be 605,278 (95% UI: 410,668 to 1,301,114) based on the proportion of foodborne cases of ascariosis, and thus DALYs being 45% (95% UI: 31 to 59) of all cases. For *E*. *multilocularis*, the equivalent calculation of DALYs associated with foodborne transmission was 312,461 (95% UI: 9,083 to 640,716), with 47% (95% UI: 4 to 75) of all cases estimated as being foodborne [4,30]. For diseases where all cases are foodborne (e.g., trichinellosis), the total number of DALYs and the number of DALYs due to foodborne transmission are equal [4].

In the crude-level estimate of a burden of 273,000 DALYs per annum attributable to foodborne transmission of ChD mentioned earlier [5], the calculation was based on the ChD burden reported in GBD, 2010 of 546,000 DALYs (95% UI: 271,000 to 1,054,000) [6], and data from a narrative review [31], in which of 959 cases, most (638) were due to oral transmission. This approximation is higher than the DALY burden estimated for many other foodborne pathogens at around the same time and included in the outputs of FERG in 2015, such as: *Bacillus cereus*, *Brucella* spp., *Clostridium botulinum*, *Clostridium perfringens*, *Echinococcus granulosus*, *Entamoeba histolytica*, *Fasciola* spp., *Giardia*, intestinal flukes, *Listeria monocytogenes*, *Opisthorchis* spp., Shiga toxin-producing *E*. *coli*, *Staphylococcus aureus*, and *Trichinella* spp. [3,4,7]. The majority of these have a global distribution and thus an even lower DALY per exposed person than foodborne ChD. More recent GBD data now available [1], give a slightly different picture—as described in Section 6.

However, extrapolation from an estimated burden of total (transmission-route independent) DALYs to the foodborne burden, based only on estimating the proportion that are foodborne is insufficient for *T*. *cruzi*. This is because for this pathogen the course and outcomes of clinical disease vary considerably according to transmission route. In particular, for foodborne transmission the rate of symptomatic acute ChD (defined as occurring relatively rapidly after infection, when trypomastigotes can be found in the circulating blood) is higher, the symptoms are more severe, there is greater cardiac involvement, and mortality is higher [21,32,33].

In general, mortality from vector-borne transmission ChD is estimated to be between 5% and 10%, whereas ChD from the foodborne infection route is estimated to be associated with 8% to 40% mortality [34], with children, pregnant or postpartum women, and the elderly accounting for most fatalities [35]. Whereas acute ChD in childhood due to vector-borne transmission often presents as a nonspecific, flu-like disease, infection via oral transmission causes more severe symptoms; fatality during acute ChD is not uncommon, particularly in children [35–38].

Comparisons of clinical signs and symptoms between ChD patients from oral infections and from vector-borne infections have been published [34,39,40] and together provide relatively clear information on important differences regarding severity of symptoms, and thus disease burden, depending on transmission route (summarized in Table 1).

**Table 1:** Summary of Chagas disease manifestation differences following vector-borne or foodborne transmission (references and greater detail included in the text below).

Disease phase	Vector-borne transmission	Foodborne transmission
Acute	Largely asymptomatic; 3% to 60% cases mild symptoms such as fever	Close to 100% experience fever—other common symptoms include myalgia, headache, and oedema
Acute	Romaña’s sign or chagoma often seen	Facial oedema in around 90% of cases
Acute	Cardiac manifestation in up to 10%, particularly in children	Early myocardial involvement occurs frequently (up to 100%)—often severe; cardiac tamponade associated with mortality
Chronic	Symptomatic phase (years or decades after infection) • For 60% to 70%: asymptomatic or indeterminate • For: 20% to 30% cardiac or digestive form (megaoesophagus/megacolon) • Both forms in 5% to 15%	Undefined—but rapid progression to long-term cardiac or gastrointestinal dysfunction indicated
**Mortality**	Estimated 5% to 10%	Estimated 8% to 40%

For vector-borne triatomine transmission, the acute phase of disease is broadly considered asymptomatic, with some nonspecific somatic signs (CDC https://www.cdc.gov/dpdx/trypanosomiasisamerican/index.html). However, between 3% and 60% of cases have been reported to exhibit some relatively mild symptoms (fever, headache, mononuclear phagocytic system involvement, and bilateral palpebral and/or leg oedema).

In contrast, in foodborne disease, acute symptomatic ChD occurs in nearly all patients, with close to 100% experiencing fever; other common symptoms include: myalgia, headache, leg and/or facial oedema, pericardial effusion, and abdominal pain [33,34,39,40]. In some cases of oral transmission, diarrhea, skin rash, dyspnoea, palpitations, hepatomegaly, splenomegaly, and hemorrhagic jaundice are also reported.

Cardiac manifestations (acute myocarditis) may occur in up to 10% of acute ChD cases with infection by the vector-borne route, particularly in children. However, in cases infected by the oral route, myocardial involvement occurs early, is often severe, and includes cardiac arrhythmias, congestive heart failure that may progress to cardiogenic shock, pericardial effusion, and pleural effusions [34]. Among patients with heart disease due to ChD, sudden cardiac death is the most common cause of death (55% to 60%), followed by heart failure (25% to 30%) [41].

Chronic ChD is generally considered to be the symptomatic phase following vector-borne transmission and may occur years or decades after initial infection. For most patients (60% to 70%) infected by the vectorial route, the chronic phase is asymptomatic or indeterminate, with amastigote invasion of smooth muscle occurring in the minority and leading to a cardiac form (20% to 30%) or a digestive form (megaoesophagus/megacolon) or mixed (both cardiac and digestive) in 5% to 15% of patients [39].

Long-term sequelae in the chronic phase of ChD following oral transmission are less well defined, but rapid progression to long-term cardiac or gastrointestinal dysfunction has been indicated [34].

## 5. Why does oral infection result in more severe symptoms?

Reasons for the differences in clinical outcome according to transmission route have been considered. One commonly cited reason is a greater parasitic load associated with oral infection compared with vectorial infection. In foodborne infection, a single triatomine crushed into a food or drink may have an infectious load of over 600,000 trypomastigotes; this is a considerably higher amount than occurs in fecal material from an infected triatomine [34,42]. In addition, a proportion of parasites entering through the skin does not survive, whereas those that are ingested may enter through cells in the mucous membrane of the oral cavity or via the stomach wall, reproducing in Peyer’s patches before migrating into the bloodstream. Both these latter routes are associated with a shorter incubation period and a higher parasite load [21]. Experimental studies in mice have shown that the 50% infectious dose (ID50) is 100-fold lower for oral challenge than for cutaneous challenge, indicating the greater efficiency of oral infection [43].

The few documented cases of meatborne infection (from ingestion of meat/blood of infected animals) also indicate greater severity in the acute phase than from vector-borne infection [20]. Interestingly, experimental infections in mice have suggested that entry of *T*. *cruzi* through the oral cavity results in more severe symptoms than through the stomach (delivered by gavage), indicating that in natural foodborne infection there are 2 entry routes that may result in different clinical responses. Differences in the mucosal pathways associated with infection site and, thus, components of the immune response, may be another reason why foodborne infection results in more severe disease than vector-borne transmission [44,45]. These authors also note that the frequent observation of facial oedema in foodborne ChD may indicate invasion already occurring in the buccal cavity.

Other considerations for why oral infection should result in greater symptom severity than infection via vector-borne infection include the association of different vectors with different transmission routes and genetic differences in *T*. *cruzi* itself.

### 5.1. Triatomine differences by transmission route

Different genera and species of triatomines can be involved in transmission of *T*. *cruzi*, with *Triatoma* spp., *Rhodnius* spp., and *Panstrongylus* spp. most associated with transmission to people [46]. For a triatomine species to be efficient for vector-borne transmission of *T*. *cruzi*, the following characteristics are important: colonizing home environments, wide distribution, anthropophily, infection and abundant excretion of infective forms, rapid defecation reflex, and colony density suitable for transmission (if a colony is very dense, smaller amounts of blood are taken, reducing post-feeding defecation and thus infection) [13]. While some of these characteristics may also be relevant for foodborne transmission (such as colonizing home environments), others are unimportant (such as rapid defecation reflex). This means that some triatomines that may be relevant to foodborne transmission are not suitable for vector-borne transmission.

Different species of triatomine that may be susceptible to *T*. *cruzi* infection may also be associated with varying likelihoods and loads of infection: for example, studies from Colombia found that the triatomine species *Panstrongylus geniculatus* was significantly more likely to be infected than other triatomine species [47] and also that *T*. *cruzi* loads in this species tended to be significantly higher than in other triatomine species, ranging from around 10^3^ to over 10^7^ parasites per ml [46]. This is particularly relevant regarding vector-borne and foodborne transmission, as *P*. *geniculatus* has a prolonged period (around 1 h) post-feeding prior to defecation. This means that this species of triatomine lacks an important characteristic (rapid defecation reflex) that makes them suitable vectors for transmission of *T*. *cruzi* through defecation into abrasions/bite wounds in the skin [2,46,48]. At the same time, it is widely distributed, occurs in a broad range of life zones, and, although inhabiting rural areas and not as highly domesticated as triatomine vectors such as *Triatoma infestans* and *Rhodnius prolixus*, is nevertheless intrusive regarding human dwellings and is attracted to artificial light [48]. In addition, although the adults are approximately 2 cm in length, the nymphs, which are also blood feeders, are much smaller, with the first instar no more than 3 mm in length, and thus easily overlooked. These factors mean that *P*. *geniculatus* may easily contaminate food or be incorporated into food that is blended—but is less likely to be involved in vector-borne transmission.

### 5.2. Genetic differences in *T*. *cruzi*

The current nomenclature of *T*. *cruzi* describes 7 distinct lineages or discrete typing units (DTUs—numbered I to VI, and TcBat) [49]. In some regions, but not all, most outbreaks of orally transmitted ChD have been associated with DTU-I [21,50]. However, other DTU have been reported from various foodborne outbreaks, including DTU-IV in Colombia [51], and also in the Brazilian Amazon [52]. This may again reflect that a wider range of vectors are associated with foodborne transmission than vector-borne transmission; investigation of 6 oral outbreaks in Colombia (among which 3 deaths were recorded) indicated that DTU-I (and DTU-IV) may be more closely associated with vectors that have a more sylvatic lifecycle (e.g., *Rhodnius pallescens* and *P*. *geniculatus*). These species are less relevant for vector-borne transmission than domesticated vectors, as explained above.

Although there have been a few reports of different pathological patterns associated with specific DTU in experimental infections in mice (e.g., [53,54]), how this information translates to human infection, and whether specific lineages are associated with the greater morbidity and mortality associated with oral infection, requires further investigation [21]. However, a study in which the *T*. *cruzi* subtype of 240 patients in Colombia with chronic ChD was investigated demonstrated that DTU-I is the predominant genotype associated with cardiomyopathy [55].

### 5.3. Treatment susceptibility

Finally, information suggests that treatment of foodborne ChD may be less successful than treatment of vector-borne ChD. Both nifurtimox (NFX) and benznidazole (BZN) have been used for >40 years for treating ChD, and current Pan American Health Organization (PAHO)/WHO guidelines [56] indicate the use of trypanocides for treating: (a) acute or congenital ChD (strong recommendation based on moderate certainty); (b) children with chronic ChD (strong recommendation based on moderate certainty); (c) girls and women of childbearing age with chronic ChD (strong recommendation based on moderate certainty); and (d) other adult patients with chronic ChD and no specific organ damage (conditional recommendation based on low certainty). However, such treatment is not recommended for adult patients with chronic ChD and specific organ damage (conditional recommendation based on moderate certainty). Although treatment has been shown to cure ChD in acute, congenital, and early chronic cases, and can improve clinical outcomes for chronic indeterminate (no organ involvement) cases [57], such treatment is not appropriate in chronic ChD with organ damage due to adverse effects and some strains of *T*. *cruzi* being resistant to these treatments (thus inspiring a hunt for therapeutic alternatives [58]).

Although it is unclear whether transmission route of infection directly or indirectly (higher parasitemia, association with particular DTU) affects therapeutic efficacy, lack of responsiveness to treatment has been reported from various foodborne outbreaks. Follow up after an outbreak in Venezuela, in which patients were treated with BZN (6 mg/kg/day) during 60 continuous days in 3 daily doses, infection evolution from acute to chronic phase in 29 patients, without seroconversion or parasitological eradication, indicated treatment failure [59]. Similarly, 10-year follow-up from another foodborne outbreak in Venezuela, in which treatment was predominantly with NFX, almost 70% of 46 patients (who had not been exposed to reinfection) remained positive [60]. An in vitro investigation on whether drug susceptibility was related to this apparent therapeutic failure indicated that phenotypic variability in the parasite population at contamination may have been a contributing factor [61].

## 6. Current estimates of burden of disease due to ChD and how to estimate the burden from foodborne ChD

A recent paper used data from the GBD Study (2019) to estimate both the global prevalence of ChD and the burden of ChD in terms of DALYs [1]. The authors report a global reduction in prevalence from 7,292,889 cases in 1990 to 6,469,283 cases in 2019, and a reduction in DALYs, with the estimates falling from 360,872 (95% UI: 153,746 to 450,827) in 1990 to 275,377 (95% UI: 184,453 to 459,354) in 2019 [1]. Results were provided by the Institute for Health Metrics and Evaluation (IHME), with ChD case definitions based on relevant International Classification of Disease (ICD)-9 and ICD-10 codes, and seroprevalence study results for nonfatal estimates. Burden was based on DALYs extracted directly from GBD, with YLL and YLD estimated by age, sex, and country, and all measures reported as counts and rates per 100,000 population (along with UI). The article notes that the GBD study estimates differ vastly from those calculated by other studies published in the literature and suggests various reasons for this. In particular, the authors highlight differences in immigration data for cases outside Latin America that may result in an underestimate in the prevalence of disease in non-endemic countries.

If these more recent GBD estimates [1] on ChD burden (275,377 DALYs) are used to deduce DALYs associated with foodborne transmission (as described in Section 3 [5]), with the same proportion considered foodborne (a conservative approximation of 50%; [5]), then we can derive a global foodborne ChD burden of around 138,000 DALYs (see Table 2). Although this is considerably lower than the previously estimated figure (273,000 DALYs [5]), based on older GBD data [6], it still exceeds those DALY burdens described in the FERG outputs of 2015 for most (11 of 15) of the pathogens listed as being exceeded in burden in Section 4 (namely: *Bacillus cereus*, *Brucella* spp., *Clostridium botulinum*, *Clostridium perfringens*, *Echinococcus granulosus*, *Fasciola* spp., *Giardia*, *Listeria monocytogenes*, Shiga toxin-producing *E*. *coli*, *Staphylococcus aureus*, and *Trichinella* spp. [3,4,7]).

**Table 2:** Estimates of DALYs associated with foodborne Chagas disease using older and newer global data, but not taking into account the greater disease severity associated with foodborne transmission.

Data estimate period	Estimated global burden (DALYs)	Suggested proportion of foodborne transmission	Estimated burden associated with foodborne transmission (DALYs)	Relevant references
Older data (2010)	546,000	50%	273,000	[5,6]
More recent data (2019)	275,377	50%	137,689	[1]

Of particular relevance in the recent ChD estimates of burden [1], is that the authors do not mention transmission route. It is not possible to determine from the IHME database whether this was considered, but this is important given that oral ChD has increasingly replaced vector-borne transmission. In addition, there should be a heavier weighting for ChD transmitted orally than via vector bite, resulting in a greater burden of disease, in terms both of YLL and YLD. This is due to the greater fatality (particularly in younger age groups) and also more serious symptoms associated with foodborne transmission.

**Fig 2:**
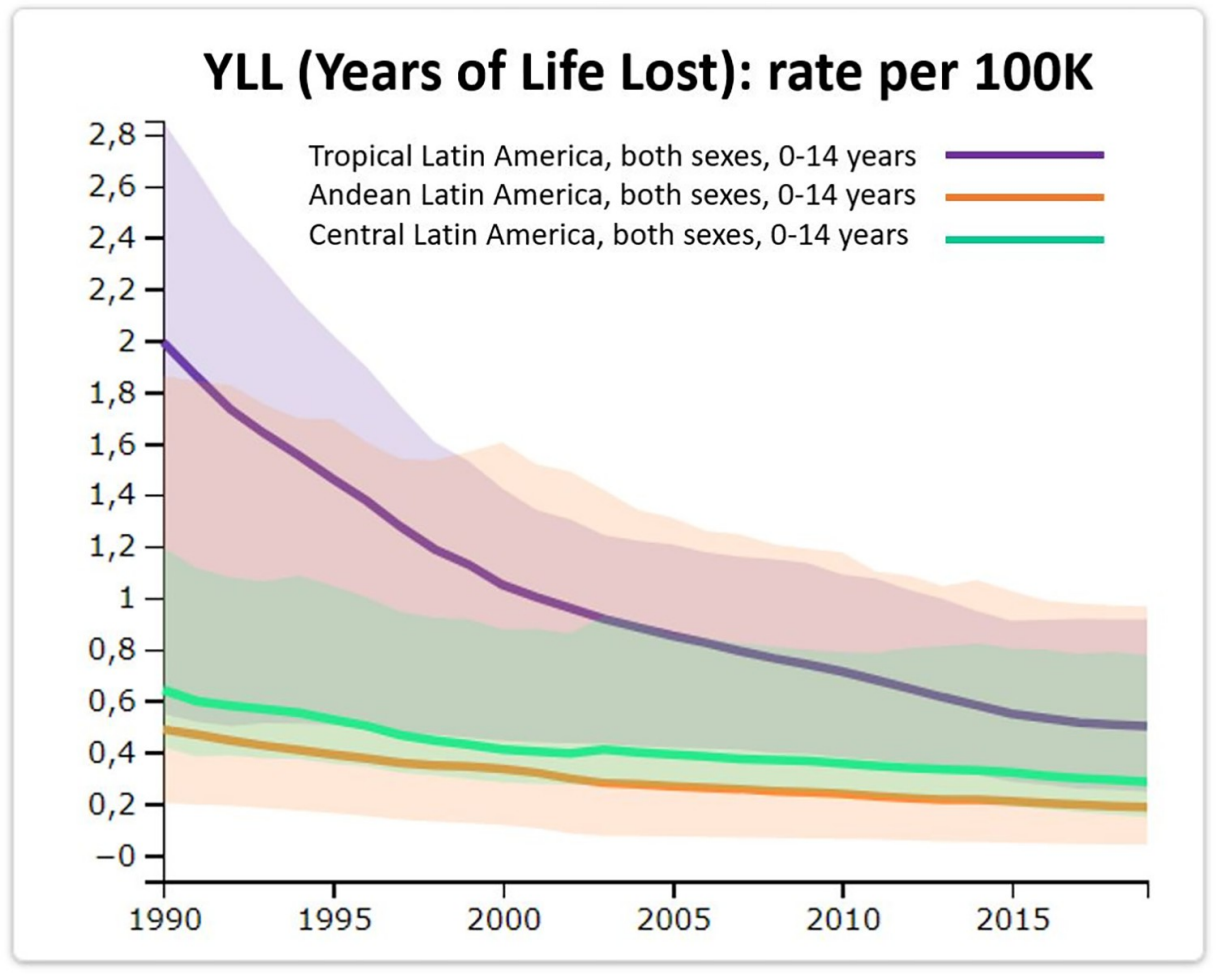
Change in rate of YLL due to ChD for children 0–14 years in 3 different regions of Latin America (shaded areas indicate uncertainty): data from IHME and graph created in Global Health Data Exchange GBD 2019 website (http://ghdx.healthdata.org/gbd-results-tool) [62]. In the absence of systematically sourced data on the proportion of foodborne ChD, whether these declines are associated with all potential exposures to ChD or primarily due to declining vector-borne disease cannot be estimated, particularly given the limited interventions to address the burden of foodborne ChD.

The data available in the IHME database do not seem to reflect this, as, for example, YLL values, have been dropping over time, in particular for younger age groups (Fig 2, [62]), especially in Tropical Latin America (Brazil and Paraguay). Although it is difficult to determine the extent of foodborne versus vector-borne burden of ChD, a synthesis of reported cases from 2002 until 2012 [31] provides 73 reports, which was double that reported in the previous decade. Among these, oral infection was the most common transmission route reported from Brazil; of 514 cases listed, 482 (94%) were described as oral transmission [31]. Similarly, the majority of reported cases from Colombia (25/49; 51%) were reported as oral transmission [31]. It has been argued that there may be more cases of vector-borne disease than foodborne disease, but foodborne cases are more visible because they result in more severe symptoms and when large outbreaks occur there is investigation and reporting bias [20]. This may be true, but as burden is linked to severity of symptoms and not only numbers of cases, and as the foodborne path may result in many cases rather than individual sporadic infections, this also contributes to the overall burden.

## 7. Conclusions

ChD remains a considerable public health issue in endemic countries of Latin America. In efforts aimed at quantifying the global health burden from foodborne diseases, it is important that it is not overlooked, simply because of its relatively restricted geographical area of endemicity. Even preliminary conservative estimates suggest that the burden from foodborne ChD is greater than for some other foodborne diseases with a global distribution. Exclusion of foodborne ChD from etiology-based burden of foodborne disease estimates may result in errors when risk ranking these diseases for the purpose of prioritizing interventions in endemic countries. Should FERG for 2021 to 2025 be able to include ChD in their estimates, then it will be essential not only to estimate the proportion of cases of ChD that are foodborne, but also to ensure that the more severe illness associated with this route of infection is taken into account.

**Learning Points**

- To date, estimates of the disease burden associated with ChD do not consider the effect of transmission route on clinical severity and disease outcome.
- ChD that has been acquired by foodborne transmission is associated with acute, as well as chronic, disease and has a different symptom spectrum than that seen in vector-borne transmission. This may be associated with actual site of infection, dose of infection, or specific genetic characteristics of the parasite that may, in turn, be related to the most likely triatomine species associated with exposure. It is unclear which of these factors is more important.
- In calculating the burden associated with foodborne ChD, it is important to bear in mind that when transmission of *T*. *cruzi* is foodborne, then the resulting disease has a higher disability weight (more severe symptoms) and case-fatality ratio than vector-borne ChD.

**Key Papers**

- Díaz-Bello Z, Alarcón de Noya B, Muñoz-Calderón A, Ruiz-Guevara R, Mauriello L, Colmenares C, Moronta E, Aponte M, Ramírez JL, Noya-González O. Ten-year follow-up of the largest oral Chagas disease outbreak. Laboratory biomarkers of infection as indicators of therapeutic failure. *Acta Trop*. 2021;**222:**106034.
- Franco-Paredes C, Villamil-Gómez WE, Schultz J, Henao-Martínez AF, Parra-Henao G, Rassi A Jr, Rodríguez-Morales AJ, Suarez JA. A deadly feast: Elucidating the burden of orally acquired acute Chagas disease in Latin America—public health and travel medicine importance. *Travel Med Infect Dis*. 2020;**36:**101565.
- Gómez-Ochoa SA, Rojas LZ, Echeverría LE, Muka T, Franco OH. Global, regional, and national trends of Chagas disease from 1990 to 2019: comprehensive analysis of the Global Burden of Disease Study. *Glob Heart*. 2022;**17(1):**59.
- Robertson LJ, Devleesschauwer B, Alarcón de Noya B, Noya González O, Torgerson PR. *Trypanosoma cruzi*: time for international recognition as a foodborne parasite. *PLoS Negl Trop Dis*. 2016;**10(6)**:e0004656.
- Velásquez-Ortiz N, Hernández C, Cantillo-Barraza O, Ballesteros N, Cruz-Saavedra L, Herrera G, Buitrago LS, Soto H, Medina M, Palacio J, González MS, Cuervo A, Vallejo G, Zuleta Dueñas L, Urbano P, Muñoz M, Ramírez JD. *Trypanosoma cruzi* parasite burdens of several triatomine species in Colombia. *Trop Med Infect Dis*. 2022;**7(12)**:445.
